# Development of an Fe_3_O_4_ Surface-Grafted Carboxymethyl Chitosan Molecularly Imprinted Polymer for Specific Recognition and Sustained Release of Salidroside

**DOI:** 10.3390/polym15051187

**Published:** 2023-02-27

**Authors:** Xingbin Ma, Shuyu Li, Jiajie Qiu, Zijie Liu, Siyu Liu, Zhifeng Huang, Yanhong Yong, Youquan Li, Zhichao Yu, Xiaoxi Liu, Hongling Lin, Xianghong Ju, A. M. Abd El-Aty

**Affiliations:** 1 College of Coastal Agricultural Sciences, Guangdong Ocean University, Zhanjiang 524088, China; 2 Zhanjiang Experimental Station, Southern-Subtropical Crop Research Institute, Chinese Academy of Tropical Agricultural Sciences, Zhanjiang 524013, China; 3 Department of Pharmacology, Faculty of Veterinary Medicine, Cairo University, Giza 12211, Egypt; 4 Department of Medical Pharmacology, Medical Faculty, Ataturk University, Erzurum 25240, Turkey

**Keywords:** drug delivery systems, salidroside, molecularly imprinted polymer, chitosan embedding Fe_3_O_4_ microspheres, drug release kinetics

## Abstract

The choice of carrier material is critical in the study of natural drug release preparations and glycosylated magnetic molecularly imprinted materials. The stiffness and softness of the carrier material affect the efficiency of drug release and the specificity of recognition. The dual adjustable aperture-ligand in molecularly imprinted polymers (MIPs) provides the possibility of individualized design for sustained release studies. In this study, a combination of paramagnetic Fe_3_O_4_ and carboxymethyl chitosan (CC) was used to enhance the imprinting effect and improve drug delivery. A combination of tetrahydrofuran and ethylene glycol was used as a binary porogen to prepare MIP-doped Fe_3_O_4_-grafted CC (SMCMIP). Salidroside serves as the template, methacrylic acid acts as the functional monomer, and ethylene glycol dimethacrylate (EGDMA) serves as the crosslinker. Scanning and transmission electron microscopy were used to observe the micromorphology of the microspheres. The structural and morphological parameters of the SMCMIP composites were measured, including the surface area and pore diameter distribution. In an in vitro study, we found that the SMCMIP composite had a sustained release property of 50% after 6 h of release time in comparison to the control SMCNIP. The total amounts of SMCMIP released at 25 °C and 37 °C were 77% and 86%, respectively. In vitro results showed that the release of SMCMIP followed Fickian kinetics, meaning that the rate of release is dependent on the concentration gradient, with diffusion coefficients ranging from 3.07 × 10^−2^ cm^2^/s to 5.66 × 10^−3^ cm^2^/s. The results of cytotoxicity experiments showed that the SMCMIP composite did not have any harmful effects on cell growth. The survival rates of intestinal epithelial cells (IPEC-J2) were found to be above 98%. By using the SMCMIP composite, drugs may be delivered in a sustained manner, potentially leading to improved therapeutic outcomes and reduced side effects.

## 1. Introduction

Molecularly imprinted materials (MIMs) have a wide range of potential applications due to their ability to selectively bind to and recognize specific target molecules. This includes extraction, isolation, purification, catalysis, and antibody simulation [1]. The use of MIMs in sustained-release drug delivery is an active area of research and development, and there is still much to be investigated about their potential in this field. As pharmacy research has become more systematic and scientific, there has been a trend toward developing drugs that are administered in smaller doses with minimal side effects and strong curative effects [2]. Most clinical applications include crude extract powder, tablets, and particles, but they have some limitations, such as the burst effect [3]. The above dosage forms and natural products often do not have a sustained release effect; they do not provide full efficacy, have a low utilization rate, and have insignificant efficacy [4]. The modern Chinese medicine approach to treating diseases involves targeting multiple aspects of the disease using multiple treatments to address the disease from various angles [5]. The primary active compounds found in *Rhodiola sacra* are salidroside, crenulatin, rhodiolosides, rhodiosin, luteolin, and catechin [6]. *Rhodiola sacra* has been shown to have anti-hypoxia, anti-fatigue, anti-aging, and anti-radiation resistance properties. In addition, it regulates the nervous, cardiovascular, cerebrovascular, and immune systems [7,8]. To control drug release and achieve targeted release of salidroside using an oligosaccharide and an imprinted drug delivery system, a new release agent that combines polymer affinity and inherent leakage needs to be developed.

To address these limitations, the use of molecularly imprinted polymers (MIPs) as drug delivery systems is a growing area of interest in contemporary pharmaceutical research [9]. Recent studies have designed new molecularly imprinted slow-release agents using chlorophyine [10], tetracycline [11], capecitabine [12], ciprofloxacin [13], and theophylline [14] as template molecules. The use of these new molecularly imprinted slow-release agents has significantly enhanced the slow-release performance, bioavailability, and effectiveness of the drugs. The release of the drug using MIPs can be controlled by factors such as heat sensitivity, acid–alkali reactions, and the presence of endogenous substances. In this context, Khadijeh et al. [15] prepared a highly selective magnetic MIP with a nuclear shell structure, which markedly increased the maximum adsorption capacity of quercetin. The sustained release mechanism observed in vitro is driven by Fickian diffusion, mainly due to the electrochemical potential gradient. Furthermore, Marcelo et al. [16] used itaconate to create a metronidazole delivery system using MIPs and found that MIP release was higher at pH 2.2 than at pH 7. Additionally, Puoci et al. [17] used sulfasalazine as a template molecule and found that the drug was not released in the stomach (pH 1.0) after oral administration but was slowly released in the intestine (pH 6.0–8.4) for targeted delivery to the colon.

Molecular imprinting involves using a drug as a template molecule to generate selective binding sites in polymers. Magnetic nanomaterials have large specific surface areas, high adsorption capacity, and superparamagnetic properties, making them easy to separate quickly. They are also environmentally friendly, simple to prepare, and have low toxicity. These features make them attractive for various applications [18]. Meloxicam and 5-fluorouracil were used as template molecules to prepare MIPs, which were functionalized using Fe_3_O_4_ nanoparticles as scaffolds [19,20]. A magnetic MIP of quercetin can be utilized as a drug delivery system. In evaluating its adsorption mechanism, the maximum capacity was determined and followed the Langmuir model. A MIP prepared using tragacanth gum as a crosslinker demonstrated excellent recognition and binding affinity for quercetin [15]. 

CC is a water-soluble derivative of chitosan with many medical effects, such as promoting wound healing [15], possessing antibacterial activity, inhibiting scars, and having analgesic effects [21]. CC has wide applications in biomedical and pharmaceutical fields due to its unique biological properties and is commonly used in pH-sensitive sustained-release drug delivery systems [22], environmental protection, and health care products [23]. CC has desirable properties, such as biocompatibility and biodegradability, making it useful as a biomaterial for wound healing and tissue engineering [24]. CC can be processed easily into nanoparticles, making it highly suitable for green chemistry and drug delivery applications. Magnetic MIPs prepared with CC have shown potential in the encapsulation and sustained release of natural small-molecule drugs, leading to improved bioavailability and efficacy.

The study aimed to optimize the synthesis of magnetic molecularly imprinted polymers (MIPs) based on the common structural properties of active compounds in *Rhodiola rosea* L. (*R. sacra*) and to develop a slow-release microsphere with high molecular recognition capability, class specificity, and selectivity. The functional monomer and salidroside ratio was optimized based on the ultraviolet spectrum, and a series of MIPs were synthesized through precipitation polymerization. The binding and release mechanism was analyzed, and the molecular recognition mechanism of polymers was explored. The optimal synthesis system of SMCMIP was selected and developed.

## 2. Experimental

### 2.1. Materials

Salidroside (purity > 98%) was obtained from Chengdu Ruiphens Biotechnology Co., Ltd. (Chengdu, China). CC (molecular weight: 240 kDa, degree of deacetylation = 90%, degree of substitution = 90%) was procured from Shanghai Macklin Biochemical Technology Co., Ltd. (Shanghai, China). FeSO_4_·7H_2_O, FeCl_3_·6H_2_O, NaOH, ethanol, ethyl orthosilicate (TEOS), and 3-aminopropyl trimethoxysilane (APTES) were purchased from Sigma-Aldrich (St. Louis, MO, USA). 1-Ethyl-3-(two methyl-aminopropyl)-carbon (EDC), 2-imine-N-hydroxysuccinimide (NHS), two ethylene glycol dimethacrylate (EGDMA), and 2,2′-azobis isobutyronitrile (AIBN) were acquired from Alfa Aesar (Heysham, UK). Porcine intestinal epithelial cells (IPEC-J2) were donated by the preventive Veterinary Medicine Laboratory of Yangzhou University. Gibco Dulbecco’s modified Eagle medium (DMEM/F12) was purchased from Invitrogen Gibco (Grand Island, NE, USA). All other materials were of analytical reagent grade and purchased from the Beijing Chemical Reagent Factory (Beijing, China).

### 2.2. Instruments and Methods

UV-Vis spectroscopy using a Nanodrop 2000C (Thermo Fisher Scientific, MA, USA) was used to determine the appropriate ratio between the dummy template and functional monomers at 275 nm. The polymers’ magnetic properties were measured at room temperature using a Vibratory Sample Magnetometer (VSM) (Quantum Design Versa lab, San Diego, CA, USA). The morphology (size, shape, and surface features) of the polymers was characterized by transmission electron microscopy (TEM; HITACHI H-7000FA, Hitachi High-Technological Corporation, Tokyo, Japan) and scanning electron microscopy (SEM; HITACHI S-4800, Hitachi High-Technological Corporation, Tokyo, Japan). The KBr compression technique and a Nexus-470 Fourier transform infrared spectrometer (FT-IR; Nicolet, MN, USA) were used to verify the infrared spectral characteristics of magnetic particles at various stages of synthesis. A high-performance liquid chromatographic system (HPLC-SY9100; Binjin, China) was used to analyze the adsorbent capacity, and HPLC-UV was used to evaluate the release ability of MIPs. Cell viability was analyzed using an enzyme-labeled instrument (MK3, Thermo Fisher, Massachusetts, USA).

### 2.3. Preparation of Fe_3_O_4_@SiO_2_-NH_2_

As previously described, magnetic Fe_3_O_4_ nanoparticles were prepared by a conventional coprecipitation method [25]. Fe_3_O_4_@SiO_2_ nanoparticles were prepared using the sol-gel method [26]. In brief, FeSO_4_·7H_2_O (0.6 g) and FeCl_3_·6H_2_O (1.6 g) were dispersed in a mixed solution of ethanol (100 mL), pure water (40 mL), and aqueous ammonia solution (10 mL) with N_2_ filling, and ultrasonically mixed for 15 min. TEOS (0.6 mL, 2.2 mmol) was then added. After stirring at 60 °C for 12 h, the obtained nanoparticles were sedimented, washed three times, and vacuum-dried for 10 h. Fe_3_O_4_@SiO_2_ (0.1 g)-activated microspheres were dispersed in 100 mL of toluene, added to 12 mL of APTES, and stirred for 5 h at 30 °C under nitrogen in a three-necked flask. The Fe_3_O_4_@SiO_2_-NH_2_ microspheres were alternately washed with dimethyl sulfoxide and ethanol three times and vacuum-dried for 12 h.

### 2.4. Preparation of Fe_3_O_4_@SiO_2_-NH_2_-CC (MCC)

MCC was prepared using the oil-coated water method. An appropriate amount of CC (0.09, 0.12, and 0.15 g) was dissolved in a water solution (4.5 mL). After dissolution, Fe_3_O_4_@SiO_2_-NH_2_ nanoparticles (0.02 g), EDC (15 μg), and NHS (5 mg) were added to a CC-aqueous solution, sonicated, and dispersed for 30 min. The mixture was slowly dropped into a solution of the emulsifier Span-80 (2 mL) and liquid paraffin (18 mL), and the reaction was stirred at room temperature for 30 min. After the reaction, formaldehyde solution (1.5 mL) was added to the mixture and stirred for 1 h. When the temperature increased to 30 °C, sodium hydroxide solution (1 mol/L) was added slowly, and the pH was 9.5. After 4 h, MCC was collected using a magnet and washed with petroleum ether and water several times. Finally, MCC was dried at −10 °C, and the yield was calculated.

### 2.5. Preparation of SMCMIP and SMCNIP

Different proportions of MAA and MCC microspheres were pre-polymerized at 4 °C. Tetrahydrofuran/ethylene glycol (1:1, *v*/*v*, 30 mL) as a porogen was polymerized at 4 °C for 30 min. Salidroside was used as a template. The crosslinkers TRIN and EDGMA, and the initiator AIBN were added to the reaction system and sonicated for dispersion and dissolution. Air was eliminated from the reaction system with nitrogen for 10 min. The water bath’s oscillometer condition was set to 60 °C and 200 revolutions for 18 h. SMCNIP was prepared using the same method without the addition of a template. After synthesis, the polymer was precipitated using a magnet. Finally, the polymers were washed with different solutions five times. The polymers were vacuum-dried at 4 °C.

### 2.6. Adsorption of SMCMIP

The hydrophilic polymer loading experiment was similar to the polymer adsorption performance experiment, and only the adsorption environment was ultra-pure water. Different concentrations of 1 mL of salidroside (0.5, 1, 2, 3, 5, 7, 9, 10, and 12 mg/mL) were prepared by dissolving 5 mg of polymer (SMCMIP and SMCNIP), and the solutions were shaken at room temperature for 2 h. When equilibrium was reached, centrifugation was carried out at high speed. The liquid supernatant was collected over a 0.22 μm filter membrane and detected by HPLC-UV. NIP was operated in the same way. The adsorption amount (*Q*, mg/mL) was calculated using Equation (1):

(1) $$Q=\frac{(C_{0}-C_{e})V}{M}$$

where *C*_0_ (mg mL^−1^) indicates the starting concentration, *C_e_* (mg mL^−1^) is the equilibrium concentration, *V* (mL) is the volume of the solution, and *M* (mg) is the polymer mass. All experiments were performed three times.

(2) $${\frac{Q}{C}}_{e}=\frac{Q_{max}-Q}{K_{d}}$$

where *Q* is the binding capacity to SMCMIP and SMCNIP, *Q_max_* is the maximum binding capacity, and *C_e_* is the equilibrium concentration of the salidroside solution. *K_d_* is the dissociation constant related to the affinity of the adsorption sites.

In addition, the selection properties of the imprinted polymers can be characterized based on the imprinting factor *I_e_* via Equation (3) as follows:

(3) $$I_{e}=\frac{Q_{SMCMIP}}{Q_{SMCNIP}}$$

where *Q_SMCMIP_* and *Q_SMCNIP_* are the partition coefficients of the imprinted and nonimprinted polymers for reaching equilibrium for target adsorption, respectively.

### 2.7. Solubility of SMCMIP

To compare the differences in the hydrophilicity of CMNIP and CMMIP, we investi-gated the swelling properties of the polymers in an aqueous solution. Approximately 50 mg of the polymer was weighed and transferred into a clean solid-phase extraction col-umn (3 mL). The total mass was obtained. Under the column with 0.9% NaCl solution at room temperature, appropriate pressure was applied to remove the excess solution in the column. After wiping the liquid on the MIP particle surface with Kimwipe paper (Kim-berly Clark Professional), the quality was monitored in real-time, and the swelling rate (SR) was calculated using the following equation:

(4) $$SR=\frac{W_{t}-W_{0}}{50}\times 100\%$$

where *W_t_* (mg) and *W*_0_ (mg) are the swollen and original polymer masses at time *t*, respectively.

### 2.8. In Vitro Drug Release

Approximately 7 mL of HCl (36.5%) and 2 g of NaCl were mixed in an appropriate amount of pure water, placed in a 1000 mL volumetric flask to constant volume, and stirred evenly via ultrasound to obtain simulated gastric juice (pH 1.5). Approximately 0.1 g of KCl, 4 g of NaCl, 3.4 g of KH_2_PO4, and 0.72 g of Na_2_HPO_4_ were weighed and dissolved in 400 mL of pure water. The pH was adjusted to 7.4, and the solution was stirred evenly via ultrasound to obtain artificial intestinal fluid.

The in vitro sustained release of salidroside from the synthesized SMCMIP loaded with the drug was investigated by immersing 5 mg of SMCMIP microspheres fully loaded with salidroside into 10 mL of simulated gastric juice (pH 1.5) and intestinal fluid (pH 7.4). Artificial liquids were prepared according to the literature [27]. The system fully oscillated at 180 rpm in a water bath oscillator at 37 °C. At 0.5–68 h, 1 mL of the supernatant was centrifuged, and 1 mL of new simulated gastric or intestinal juice was added to continue the experiment. Analysis and detection were carried out through HPLC-UV. CMNIP was used as a control experiment in the same way. The cumulative release (CR) was calculated using the following equation:

(5) $$CR=\frac{10C_{n}+\sum_{}^{}C_{\left(n-1\right)}}{W_{0}}\times 100\%$$

where *W*_0_ (mg) represents the weight of salidroside adsorbed on the polymer, and *C_n_* (ng mL^−1^) and *C* (*n* − 1) (ng mL^−1^) refer to the concentration of salidroside in the solution taken at *n* and *n* − 1 times, respectively. The constant 10.0 refers to the total volume, and 1.0 represents the volume of the fresh solution.

### 2.9. In Vitro Release Kinetics

To study the sustained release mechanism, two models were used to compare and relate some release parameters; the kinetic study of drug release is often helpful in obtaining physically meaningful parameters [15]. Therefore, the diffusion properties and release kinetics of SMCMIP and SMCNIP were investigated using mathematical models, such as Higuchi and Korsmeyer-Peppas, which are expressed in the following equations:

(6) $$\frac{M_{t}}{M_{\infty }}=At^{1/2}$$

where *A* is the Higuchi constant, which reflects the formula’s characteristics, and *M_t_*/*M*_∞_ is the drug release at a small fraction of time *t*. According to this model, mapping *M_t_*/*M_∞_* with *t*^½^ will lead to a straight line if the matrix is based on a diffusion-based drug release mechanism.

(7) $$\frac{M_{t}}{M_{\infty }}=Kt^{n}$$

where *K* is the kinetic constant and *n* is an exponential that describes the release mechanism. *M_t_* is the amount of release at time *t*, and *K* is the release rate constant.

### 2.10. Proliferation Test on Intestinal Epithelial Cells (IPEC-J2)

Cell counting kit-8 was used to detect cell proliferation. IPEC-J2 cells in the logarithmic growth phase were inoculated into 96-well plates (density 1 × 10^6^ cells/well) at 37 °C with 5% CO_2_. After the cells adhered to the wall, SMCMIP (200, 400, 600, 800, 900, and 1000 μg mL^−1^) was added for 24 h. At the same time, blank and negative groups (SMCMIP and SMCNIP) were established. Each treatment group was set with six replicates. After 24 h, 10 μL of CCK8 solution was added to each well and incubated in CO_2_ for 2 h. Finally, the OD was measured in each well by using an enzyme-labeled instrument at 450 nm.

## 3. Results and Discussion

### 3.1. Synthesis of SMCMIP

**Figure 1:**
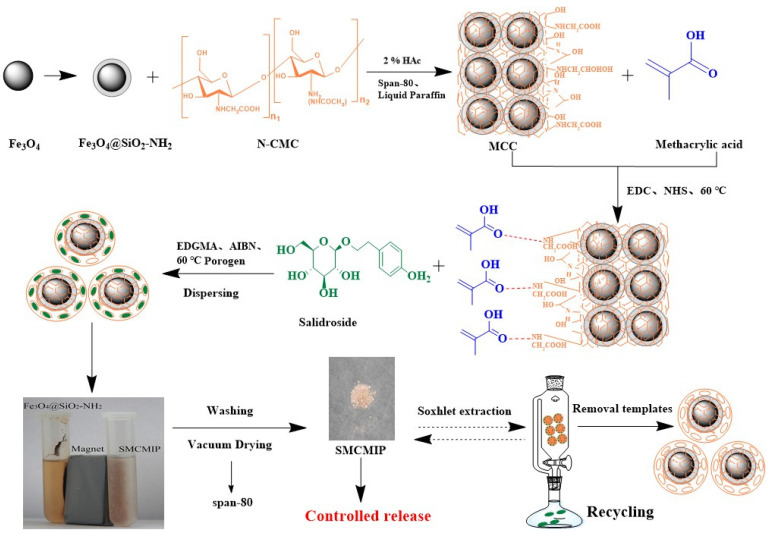
Scheme representing the procedure for preparing SMCMIP.

**Figure 2:**
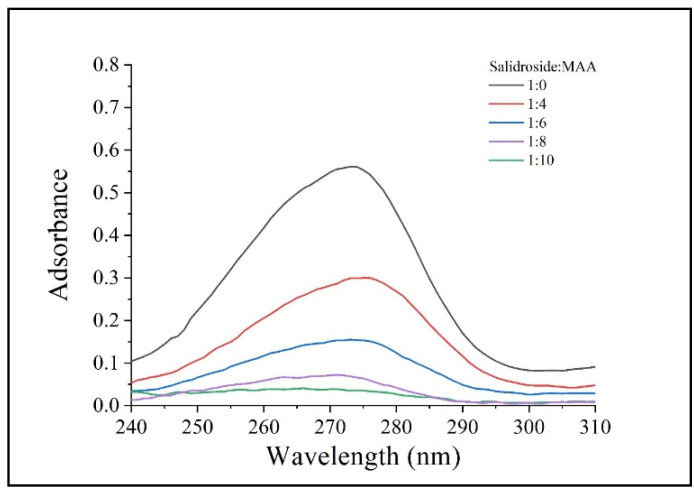
UV spectra of salidroside with MAA at different molar ratios.

The synthesis of SMCMIP and polymer and the corresponding release performance are shown in Figure 1. The properties of the imprinting polymers were compared with those of TRIM and EGDMA as crosslinkers. The polymer yield using EGDMA as a crosslinker was higher than that using TRIM under the same reaction conditions. The swelling rate of SMCMIP was 672% ± 2%, as shown in Table 1. The moderate size of the CC molecules on Fe_3_O_4_@SiO_2_ with many hydrophilic groups could be the cause. The different ratios between the template molecule and functional monomers were examined using UV spectra. The optimal molar ratio of the template with the functional monomer was 1:4 (Figure 2). Based on this finding, the preparation of SMCMIP was successful.

### 3.2. Characterization of SMCMIP

**Figure 3:**
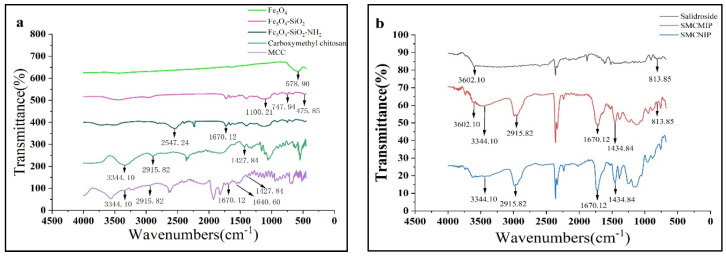
FTIR spectra of Fe_3_O_4_, Fe_3_O_4_@SiO_2_, Fe_3_O_4_@SiO_2_-NH_2_, CC, and MCC (**a**); salidroside, SMCMIP, and SMCNIP (**b**).

In the prepared functional scaffold part, the OH group on Fe_3_O_4_@SiO_2_ may react with the carboxyl group on CC to form an unstable intermediate. After dehydration, the reaction with CC continued to produce SMCC. The success of the synthesis of the functional monomer was determined based on the FT-IR spectra (Figure 3a). The peaks at a specific frequency of 579 cm^−1^ are the characteristic absorption peaks of Fe-O-, and the peaks at 1100.21, 747.94, and 476 cm^−1^ could be attributed to Si-O-Si. These peaks were interpreted as evidence of the successful wrapping of SiO_2_ around the Fe_3_O_4_ nanoparticles. The peak observed at 2547 cm^−1^ is attributed to the extended vibrational peaks of the N-H bond in Fe_3_O_4_-SiO_2_-NH_2_. Meanwhile, additional peaks are observed in the FT-IR spectra. The peak at 1670 cm^−1^ is attributed to the C=O group. The peak at 1428 cm^−1^ is attributed to the symmetric stretching vibration of the -COO- group. The peaks at 2916 cm^−1^ and 3344 cm^−1^ are attributed to the -C-O-C- and -NH2 groups, respectively. Compared to CC, a peak was observed at 1641 cm^−1^. This peak is attributed to the stretching vibration of the C=N group and is unique to the magnetic chitosan microspheres [28]. The symmetric stretching vibration peaks of -COO^-^ appeared at 1428 cm^−1^, and -NH_2_ and C=O peaks were observed at 3344 and 1670 cm^−1,^ respectively (Figure 3a). Consequently, it can be inferred that Fe_3_O_4_ magnetic nanoparticles were successfully encapsulated within CC.

In the FT-IR spectrum (Figure 3b), 3602 cm^−1^ and 814 cm^−1^ are the characteristic peaks of salidroside, and the stepped peaks are in the range of 1400 to 1000 cm^−1^. Symmetric stretching vibration peaks of -COO- are observed at 1428 cm^−1^, and peaks at 3334, 2916, and 1670 cm^−1^ can be attributed to MCC, namely, the extended vibration absorption peaks of N-H, -C-O-C- and C=O. Meanwhile, salidroside peaks were also observed in SMCMIP. Thus, the synthesis of SMCMIP is inferred.

**Figure 4:**
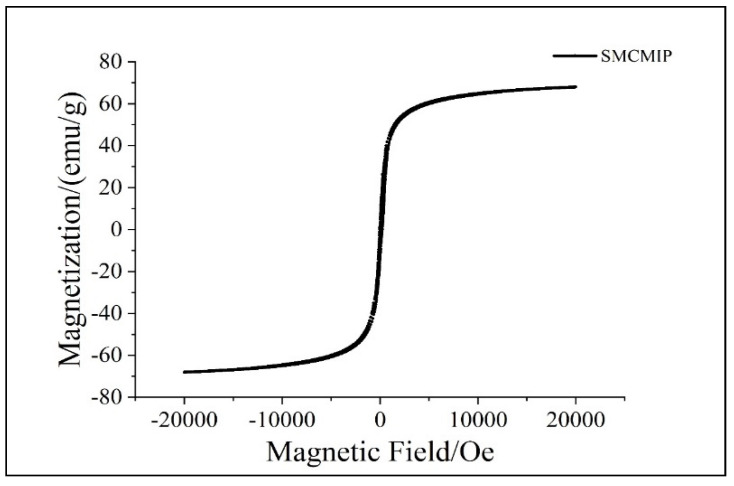
Magnetization curves obtained by vibrating sample magnetometer (VSM) at room tem−perature: SMCMIP.

Magnetic MIPs have small particle sizes, strong magnetic properties, and stable nature, making them useful for various applications. As the shell structure increased, the magnetic field strength weakened and eventually reached a saturation state, suggesting changes in the magnetic properties of the material as a result of its structure, as shown by VSM (Figure 4). The saturated magnetic strength of the different levels was 70.42 emu g^−1^. The S-shaped hysteresis loop, small residual magnetic force, and moderate coercive force also suggest that SMCMIP has strong magnetic properties and can retain its magnetism over time. The magnetic field caused the MIPs to be uniformly dispersed in the solvent and quickly absorbed into the bottle wall, which took only 1.5 min. These results showed that SMCMIP exhibited good magnetic properties and effective adsorption.

### 3.3. Morphology of SMCMIP by SEM

**Figure 5:**
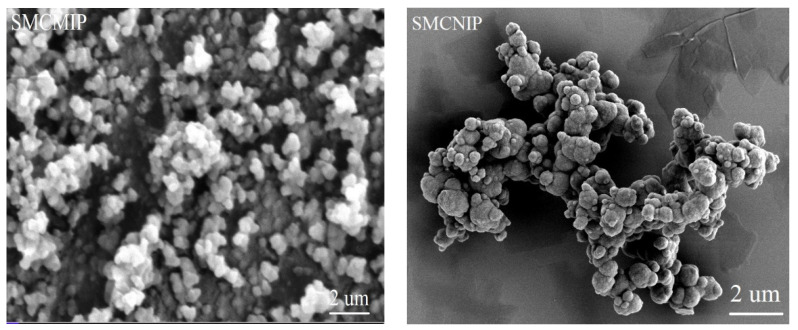
Scanning electron microscopy images of SMCMIP and SMCNIP.

Template molecules can play a role in determining the particle size and surface morphology of the final MIP products [29,30]. Compared with SMCNIP, the surface roughness of SMCMIPs could be due to the imprinting of the template molecules, leading to a higher specific surface area, improved stability, and higher selectivity for the target analyte (Figure 5). The shape of SMCMIP was more rounded and easier to disperse than that of SMCNIP, indicating that the template molecule had an impact on the growth and dispersion of the spherical particles during synthesis. SMCMIP was a homogeneous monodisperse particle with a particle size of approximately 460 nm. The SMCNIP surface was smooth, whereas the SMCMIP surface was rough. Additionally, the average particle size of SMCMIP was smaller than that of SMCNIP, indicating that the template molecule-imprinted polymers had a large surface area layer, and the imprinted polymer was coated on the surface of SMCMIP.

### 3.4. Adsorption Isotherms of SMCMIP

**Figure 6:**
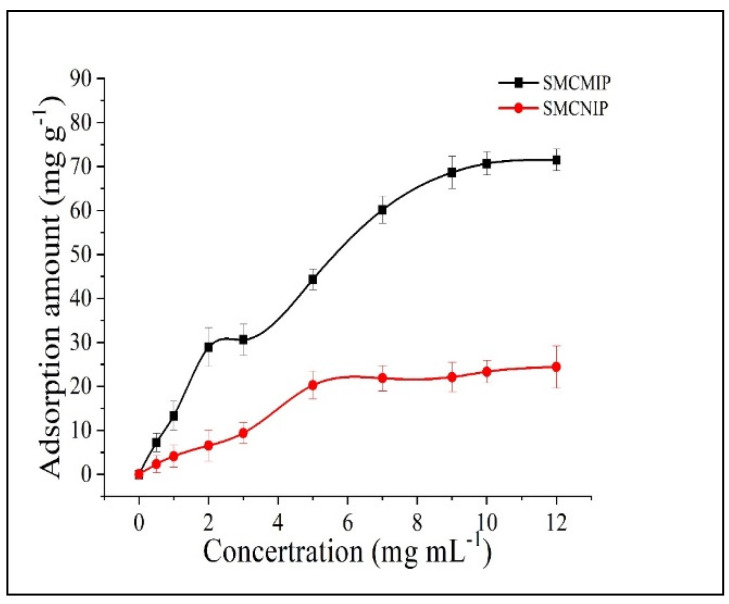
Adsorption amounts of SMCMIP and SMCNIP with different concentrations of salidroside.

**Figure 7:**
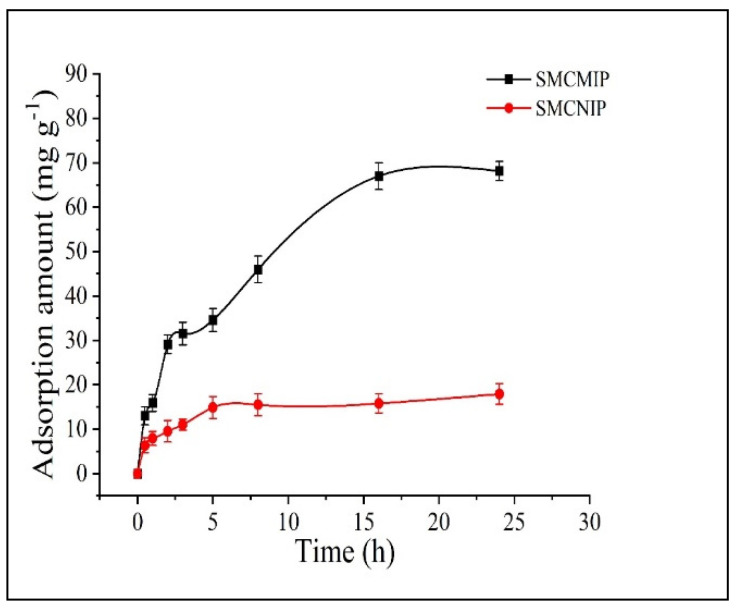
Kinetic curves of salidroside on SMCMIP and SMCNIP.

To study the binding properties of the polymer, we measured the binding amount of SMCMIP and SMCNIP at room temperature after the adsorption reached equilibrium, and the equilibrium concentration (*C_e_*) was illustrated. As shown in Figure 6, when the concentration exceeded 5 mg/Ml, the adsorption amount of SMCMIP to salidroside was significantly higher than that of SMCNIP, but SMCMIP had low adsorption and was nonselective for crenulatin, which exists in *R. sacra*. The *I_e_* value was 1.12. The adsorption concentration of SMCMIP still increased, indicating that beyond a certain concentration, the adsorption capacity of SMCMIP was mainly derived from the specific binding site. In contrast, the adsorption of SMCNIP involved nonspecific binding and was irregular. The adsorption of salidroside by SMCMIP increased with time and was balanced after 20 min (Figure 7). SMCNIP reached equilibrium after 5 min. Scatchard analysis was performed, and SMCMIP demonstrated a specific adsorption process. The adsorption capacity could be mainly attributed to the two specific binding sites. The maximum adsorption capacity (*Q_max_*) values were 130 and 371 mg g^−1^, whereas the adsorption of SMCNIP was nonspecific and irregular.

**Figure 8:**
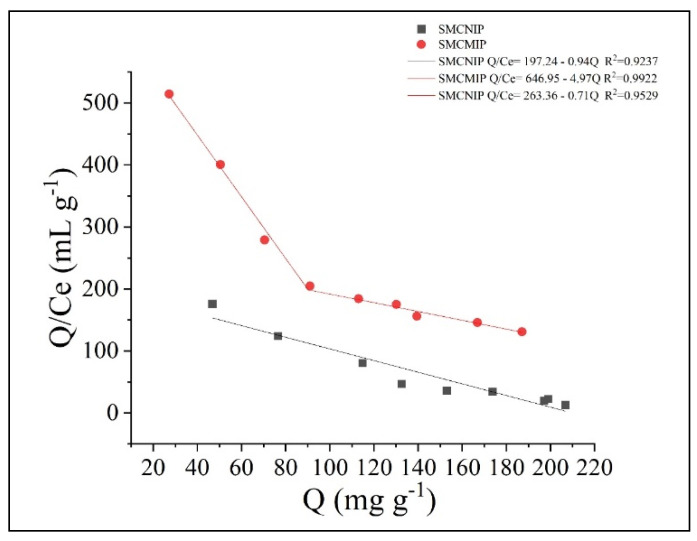
Scatchard plots for SMCMIP and SMCNIP.

For the binding amount of the target molecule and its analogs (*Q*), the binding properties of the imprinted polymer to the template molecules were studied using the Scatchard equation. By *Q*/Ce mapping to *Q* of salidroside, *Q_max_* of the polymer sample was obtained, where *Q* is the adsorption amount; *C_0_* and *C_e_* are the sample concentrations in the solution before and after adsorption, respectively; and *K_d_* is the dissociation constant. In Figure 8, the two straight lines with a clear nonlinear relationship indicated that the binding sites of the imprinted polymer were not entirely equivalent for the imprinted molecules, and two different binding sites were present. Fitting of the two-segment linear parts of the figure yielded the fitting of the linear equations, namely, the high- and low-affinity site equations.

### 3.5. In Vitro Sustained Release of SMCMIP

**Figure 9:**
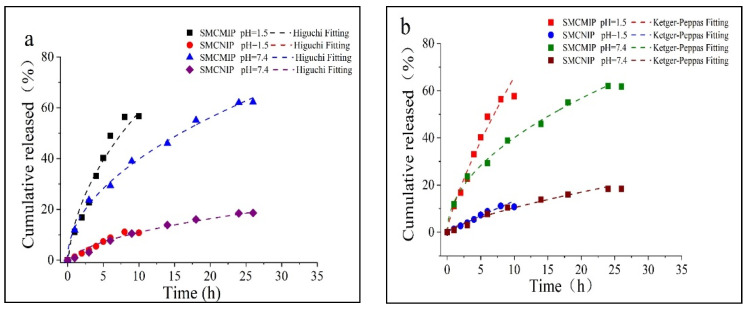
Cumulative release and fits of salidroside from SMCMIP and SMCNIP in simulated gastric/intestinal fluid (at (**a**): 25°C, (**b**): 37 °C).

For SMCMIP, the salidroside release amount was faster in the simulated gastric juice (reaching 50% at 6 h) than in the intestinal fluid (18 h near or less than 50%), as shown in Figure 9a,b. However, the salidroside release performance of SMCNIP was not sensitive to Ph changes. The rapid release rate in acidic Ph could be attributed to the high solubility of the drug at this Ph. The affinity of salidroside for the SMCMIP cavity was weakened because the hydrogen ions in the release medium had to compete with the acrylic group (pKa = 4.5). These factors promoted the rapid release of the drug under acidic conditions. In the simulated gastric juice (Ph 1.5), the polymer hydroxyl groups derived from salidroside and CC were ionized. The high ionic strength overcame the electrostatic interaction between the salidroside and the polymer to promote the release. However, some ionic effects could be expected with the drug’s affinity for CC, providing weak control over the matrix release process in a simulated fluid at Ph 7.4. In this group, the salidroside release rate of CC in the reticular polymer met the sustained release requirement. After the analysis and determination of the vehicle SMCMIP and SMCNIP at 25 °C and 37 °C, the amount of salidroside in the extract of each sample was measured to be approximately 77% and 86%, respectively. Compared with SMCNIP, 37 °C was more favorable for salidroside release, in which the release rates reached 86% and 77%, possibly because loaded SMCMIP was more favorable for a specific release; thus, the novel SMCMIP is a potential candidate for the continuous release of salidroside. The release rate of salidroside can be used to control the release time of the drug, thereby controlling the effect of the application.

Fick proposed that the flow of diffusion material through the unit cross-sectional area perpendicular to the diffusion direction in unit time is proportional to the concentration gradient at that section; that is, the greater the concentration gradient, the greater the diffusion flux. According to Fick’s first law [30], the diffusion coefficients of SMCMIP ranged from 3.07 × 10^−2^ cm^2^/s to 5.66 × 10^−3^ cm^2^/s. Depending on the ratio of SMCMIP and SMCNIP diameters to the thickness and the spherical structure with an uneven particle size distribution, *n* was correlated with the release index. Correlation studies confirmed that for a globular structure, *n* < 0.45; for pure Fick diffusion, 0.45 <*n* < 0.89; and for the Fick diffusion and anomalous mechanism, *n* > 0.89 [20]. In the mock gastric juice, SMCMIP was dominated by Fick diffusion. Anomalous diffusion was predominant in the simulated intestinal fluid in Table 2.

### 3.6. Determination of the Effect of SMCMIP and SMCNIP on IPEC-J2 Cell Viability by CCK8

**Figure 10:**
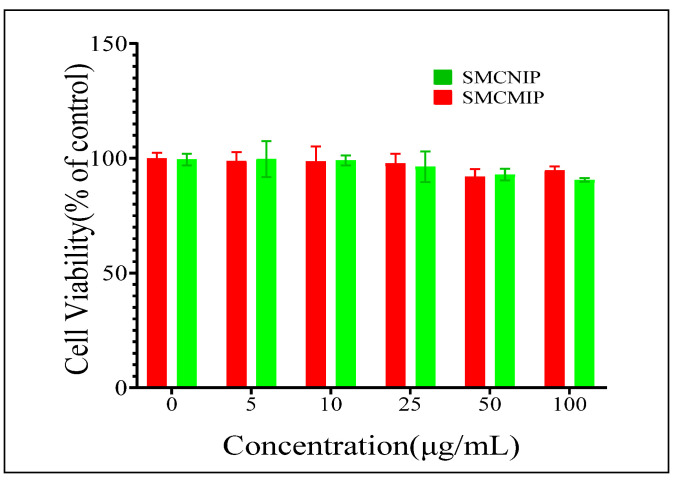
Effect of SMCMIP and SMCNIP on IPEC-J2 cell viability.

The cytotoxicity of biomaterials is an important factor in measuring the future applications of the material; as a safe and effective drug vehicle, the material itself should be nontoxic to cells. Therefore, we first investigated normal IPEC-J2 cells by using the CCK8 assay. As shown in Figure 10, after 72 h, the survival rate of cells cultured in SMCMIP and SMCNIP solution was approximately 98% ± 3%, proving that SMCMIP exhibited good biocompatibility and no cytotoxicity; thus, SMCMIP could be used as a drug carrier.

## 4. Conclusions

In conclusion, a novel magnetic surface MIP with nuclear shell morphology was synthesized for the selective recognition of salidroside with Fe_3_O_4_@SiO_2_ nanoparticles grafted with CC as the magnetic core, MAA as a functional monomer, MCC as a scaffold, and EGDMA as a crosslinker. The maximum theoretical adsorption capacities of salidroside were 130.17 and 370.93 mg g^−1^. In all cases, the imprinted magnetic high affinity, specific binding of selectivity, and adsorption kinetic rates were high for template quality control with an integrated platform. Synthetic SMCMIP with excellent biocompatibility, such as easy preparation, fast mass transfer rate, satisfactory adsorption capacity, easy separation, and specific identification capabilities, was obtained. SMCMIP could be useful for targeted drug delivery. However, several factors need to be considered when using Fe3O4-grafted CC for drug delivery, such as the drug particle size and shape, the drug release rate, and the stability of the drug-polymer complex. Further research is therefore needed to fully evaluate the potential of this polymer for drug delivery applications.

## Figures and Tables

**Table 1:** Composition of polymers.

Microspheres Name	Template (Salidroside)	Functional Monomers (MAA)	Functional Scaffold	Cross-Linker	Salidroside: MAA Molar Ratio	Yield (%)	Saturated Swelling (%)
SMCNIP	-	34.44 mg	MCC (108.7 mg)	EGDMA (396.4 mg)	-	62	44 ± 6
SMCMIP	30.03 mg	34.44 mg	MCC (108.7 mg)	EGDMA (396.4 mg)	1/4	74	67 ± 2
SMCMIP_1_	30.03 mg	34.44 mg	MCC (108.7 mg)	EGDMA (396.4 mg)	1/2	12	33 ± 3
SMCMIP_2_	30.03 mg	34.44 mg	MCC (108.7 mg)	EGDMA (396.4 mg)	1/6	24	30 ± 4
SMCMIP_3_	30.03 mg	34.44 mg	MCC (217.4 mg)	TRIM (396.4 mg)	1/4	24	29 ± 4

**Table 2:** Fitting results of SMCMIP and SMCNIP release curves.

Sample	pH	Higuchi		Korsmeyer-Peppas		Release Mechanism
		Fits Equation	R^2^	Fits Equation	R^2^	
SMCMIP	1.5	M_t_ = 21.47 t^1/2^ − 7.69	0.9422	M_t_ = 11.19 t^0.77^	0.9840	Anomalous
	7.4	M_t_ = 12.72 t^1/2^ − 0.13	0.9959	M_t_ = 12.56 t^0.43^	0.9941	Fickian
SMCNIP	1.5	M_t_ = 4.33 t^1/2^ − 2.17	0.9053	M_t_ = 1.54 t^0.94^	0.9890	Anomalous
	7.4	M_t_ = 4.15 t^1/2^ − 2.14	0.9646	M_t_ = 2.01 t^0.32^	0.9650	Fickian

## Data Availability

The data used to support the findings of this study are available from the corresponding author upon request.
